# Identification of an XRCC1 DNA binding activity essential for retention at sites of DNA damage

**DOI:** 10.1038/s41598-019-39543-1

**Published:** 2019-02-28

**Authors:** Mac C. Y. Mok, Anna Campalans, Monica C. Pillon, Alba Guarné, J. Pablo Radicella, Murray S. Junop

**Affiliations:** 1 Department of Biochemistry and Biomedical Sciences, McMaster University, Hamilton, Ontario L8S4K1 Canada; 2 Institute of Cellular and Molecular Radiobiology, CEA, UMR967 INSERM, F-92265 Fontenay aux Roses, France; 3 Department of Biochemistry, Schulich School of Medicine and Dentistry, Western University, London, Ontario N6A5C1 Canada; 4 Present Address: Department of Biochemistry, Schulich School of Medicine and Dentistry, Western University, London, Ontario N6A5C1 Canada

## Abstract

Repair of two major forms of DNA damage, single strand breaks and base modifications, are dependent on XRCC1. XRCC1 orchestrates these repair processes by temporally and spatially coordinating interactions between several other repair proteins. Here we show that XRCC1 contains a central DNA binding domain (CDB, residues 219–415) encompassing its first BRCT domain. In contrast to the N-terminal domain of XRCC1, which has been reported to mediate damage sensing *in vitro*, we demonstrate that the DNA binding module identified here lacks binding specificity towards DNA containing nicks or gaps. Alanine substitution of residues within the CDB of XRCC1 disrupt DNA binding *in vitro* and lead to a significant reduction in XRCC1 retention at DNA damage sites without affecting initial recruitment. Interestingly, reduced retention at sites of DNA damage is associated with an increased rate of repair. These findings suggest that DNA binding activity of XRCC1 plays a significant role in retention at sites of damage and the rate at which damage is repaired.

## Introduction

The repair of modified DNA bases and single strand breaks via the base excision repair (BER) and single strand break repair (SSBR) pathways requires highly coordinated repair events that are mediated by numerous protein-protein interactions involving XRCC1 [reviewed in^1,2^]. Cells lacking XRCC1 experience increased sensitivity to alkylating agents^3^ and high levels of sister chromatid exchanges^4^. In mice, disruption of *Xrcc1* results in early embryonic lethality^5^. Moreover, the lack of XRCC1 is associated with poor clinical outcome in breast cancer patients^6^ and PARP1 hyperactivation-linked cerebellar ataxia^7^.

XRCC1 is thought to facilitate the multi-step BER/SSBR process through coordinated protein-protein interactions that rely on its modular domain organization. XRCC1 contains three distinct domains (the N-terminal domain, and two independent BRCT domains), connected by two intervening unstructured linkers, which serve as critical interacting platforms for various repair factors^8^. Well documented XRCC1 interactions include: the N-terminal domain (NTD, residues 1–160) with Polβ^9,10^; the first BRCT domain (BRCT1; residues 301–415) with PARP1, APE1, MPG, NTH1 and NEIL1/2^11–13^; and the second BRCT domain (BRCT2; 534–633) with DNA Ligase3^14^. In addition, flexible linker regions also mediate protein-protein interactions, with a first linker binding REV1^15^, hOGG1^16^, NTH1 and NEIL2^13^, and a second linker interacting with APTX, APLF and PNKP^17–19^. Determining the precise functional consequences of these interactions remains an important area of investigation within single strand break and base excision repair.

Although XRCC1 persists at sites of DNA damage over the entire course of repair, whether its retention is dependent on protein-protein interactions or direct association with DNA has not been determined. The N-terminal domain of XRCC1 has been reported to bind DNA *in vitro* with a preference for damaged forms of DNA, specifically those harbouring nicks and gaps^20,21^. Weak DNA binding (dependent on chemical crosslinking) has also been observed for both BRCT domains^22,23^; however, the implications of XRCC1’s DNA binding activity on recruitment and retention to damage sites, and subsequent repair, have not been determined.

In this study, we systematically characterized regions of XRCC1 for DNA binding activity. A segment of XRCC1 encompassing the first BRCT domain and preceding N-terminal linker was shown to retain all necessary determinants for DNA interaction. Point mutants within this central DNA binding domain (CDB) that selectively disrupt DNA interaction were identified and used to evaluate the functional importance of XRCC1-DNA binding in cells. Findings presented here indicate that DNA binding activity of XRCC1 is dispensable for initial recruitment to sites of DNA damage, but vital for retention and ability to form stable repair foci. Interestingly, reducing retention of XRCC1 at sites of DNA damage results in a significant increase in the rate of repair.

## Material and Methods

### Vector construction

The human XRCC1 gene was acquired from Open Biosystems (clone ID 4646806, accession BC023593). Gateway cloning (Invitrogen) was used to generate bacterial expression constructs of full length XRCC1, XRCC1^1–183^, XRCC1^219–415^, XRCC1^301–415^, XRCC1^219–300^ and XRCC1^521–633^. Primers used in PCR reactions for vector construction are listed in Supplementary Fig. S1. PCR products were first moved into a pDONR201 entry vector (Invitrogen) and subsequently recombined into destination vectors, pDEST15 (Invitrogen) for full length XRCC1; pDEST544 (Addgene 11519) for XRCC1^219–415^ and XRCC1^219–300^; and pDEST17 (Invitrogen) for XRCC1^1–183^ and XRCC1^301–415^. All constructs were designed to include a TEV protease cleavage site between the N-terminal fusion and XRCC1 coding region. An additional C-terminal hexa-histidine tag was added to full length XRCC1 to improve recovery of full length protein. XRCC1^219–633^ was cloned into a pLic-His vector (kind gift from Stephen Bottomley of Monash University, Australia^24^) using ligation independent cloning (LIC) and CloneEZ enzyme (Genescript). For cell-based functional studies, XRCC1 was fused to YFP in pEYFP-N1 (Clontech) as previously reported^25^. A C-terminal NLS was added following YFP using overlapping PCR (primers listed in Supplementary Fig. S2). To generate WT and P1/3 XRCC1^219–633^-YFP-NLS constructs for bacterial expression, Gateway cloning (Invitrogen) was used as described above with XRCC1 in pEYFP-N1 as the template (primers listed in Supplementary Fig. S1).

Mutagenesis was performed using the one-step site-directed deletion, insertion, single and multiple-site plasmid mutagenesis protocol described by Liu *et al*.^26^. Primers used for mutagenesis are listed in Supplementary Fig. S2. All vector constructs and mutations were confirmed by DNA sequencing covering the entire open reading frame.

### Protein purification

All proteins were expressed in Rosetta (DE3) pLysS cells (Novagen). Cells were grown in 4 L of LB supplemented with ampicillin (0.1 mg/mL) at 37 °C to an OD_600_ of 0.5 and protein expression induced with 1 mM IPTG at 20 °C 16 hr. XRCC1^1–183^ was auto-induced at 16 °C over two days. Cells were harvested by centrifugation at 3,315 × g for 15 min and re-suspended in NiA buffer (20 mM Tris pH 8, 500 mM KCl, 3 mM 2-mercaptoethanol, 10% (v/v) glycerol, 10 mM imidazole) prior to lysis by sonication. The lysate was clarified by centrifugation at 48,384 × g for 45 min and soluble protein applied to an IMAC 5 mL column (GE Healthcare). Bound protein was washed with NiA buffer containing 10 mM imidazole (20 column volumes) followed by 40 mM imidazole (10 column volumes) before elution with 300 mM imidazole. Eluted protein was digested with TEV protease under standard conditions to remove affinity tags. Except for XRCC1^1–183^, which was immediately re-run on an IMAC column, cleaved protein was further purified using ion exchange chromatography (MonoQ, GE Healthcare) for full length XRCC1, XRCC1^219–633^ and XRCC1^521–633^ whereas XRCC1^219–300^, XRCC1^219–415^ and XRCC1^301–415^ were purified using a MonoS column (GE Healthcare). In all cases protein was eluted over a linear salt gradient (0–500 mM KCl) with buffer containing 20 mM Tris pH 8.0 (for MonoQ) or HEPES pH 8.0 (for MonoS) and 3 mM 2-mercaptoethanol. Except for full length XRCC1, which contained a C-terminal His tag, fractions containing XRCC1 were pooled and further purified by collecting the unbound fraction from running an additional IMAC column. A final size exclusion chromatography step was required for full length XRCC1. Purified proteins (Supplementary Fig. S3) were buffer exchanged (20 mM Tris pH 8.0, 200 mM KCl, 3 mM 2-mercaptoethanol, 10%(v/v) glycerol), concentrated by ultrafiltration (Corning) and stored at −80 °C.

### DNA substrates

The design of DNA substrates followed those reported by Marintchev *et al*.^21^. DNA oligonucleotides were purchased from Integrated DNA Technologies. DNA substrates used for binding studies were generated by annealing complementary oligos (Supplementary Fig. S4). The bottom 39 base strand was 3′ end-labelled with 6-FAM for detection. Oligos were dissolved in water and annealed using a thermocycler (Thermo Scientific) with a temperature gradient from 100 to 25 °C and cooling rate of 1 °C/min. Annealed oligos were purified using a 1 mL MonoQ ion exchange column (GE Healthcare), equilibrated with 20 mM Tris pH 8.0 and resolved with a gradient of 0–1 M KCl. Purified DNA substrates were buffer exchanged (10 mM Tris pH 8.0, 1 mM EDTA) using ultrafiltration (Nanosep, Pall Corporation) and stored at −20 °C.

### DNA binding assay

Electrophoretic mobility shift assays were performed to assess the extent of protein-DNA interaction. Prior to addition of protein, the reaction mixture (18 µL) contained DNA substrate (5 nM) in binding buffer (10 mM Tris pH 8, 100 mM NaCl, 3 mM MgCl_2_, 1 mM EDTA, 0.1% CHAPS, 10 mM 2-mercaptoethanol, 7.5% glycerol, 0.02 mg/mL bovine serum albumin). Protein (2 µL) was added to the reaction mixture to a final volume of 20 µL resulting in a final buffer composition containing 12 mM Tris pH 8.0, 100 mM NaCl, 20 mM KCl, 3 mM MgCl2, 1 mM EDTA, 0.1% CHAPS, 10 mM 2-mercaptoethanol and 8.5% glycerol. Reactions were further incubated at room temperature for 1 hr. Electrophoresis was performed using discontinuous native PAGE gels prepared with 4% and 15% polyacrylamide stacking and resolving regions, respectively. The stacking portion was buffered with 140 mM Tris pH 8.0 while the resolving portion contained 45 mM Tris pH 8.0, 45 mM boric acid, 1 mM EDTA. A discontinuous gel was used to help sharpen bands. Electrophoresis was performed at room temperature and constant voltage (10 V/cm) for 90 min. Gels were imaged using a ChemiDoc (Bio-Rad) with settings optimized for fluorescein detection and the degree of DNA binding quantified using ImageJ. Binding curves for each XRCC1 variant were generated by monitoring the disappearance of unbound 39 bp DNA substrate as a function of increasing XRCC1 concentration. Unbound substrate was quantified and compared to a DNA-only control to determine the fraction of unbound substrate. Each measurement was performed in triplicate. Average values were imported into Sigmaplot 12 and regression analysis performed. The best curve fitting was attained using the 3 parameter Hill equation function: $f=\frac{a{x}^{b}}{{c}^{b}+{x}^{b}}$ where f is the fractional occupancy, x is the free ligand concentration, a is the maximum y-axis value, b is the Hill coefficient and c is the protein concentration where 50% of DNA is in complex (i.e. equilibrium dissociation constant).

### Small Angle X-ray Scattering

XRCC1^219–415^ was buffer exchanged using a size exclusion column (Superdex 200; GE Healthcare) equilibrated with 20 mM Tris pH 8.0, 100 mM KCl, 3 mM 2-mercaptoethanol. This buffer was chosen to minimize solution scattering while maintaining protein stability. This buffer had no significant impact on DNA binding compared to buffers used for EMSA analysis (Supplementary Fig. S5). 39 base-pair duplex DNA (without fluorescent tag) described in (Supplementary Fig. S4) was buffer exchanged into 20 mM Tris pH 8.0, 100 mM KCl, 3 mM 2-mercaptoethanol using a 3k Da MWCO nanosep ultrafiltration column (Pall Corporation). Samples were centrifuged at 10,000 × g for 10 min to remove any particulates from solution and sample homogeneity was confirmed by dynamic light scattering. Scattering data for XRCC1 alone was measured at concentrations of 266, 201, 187 and 93 µM in 20 mM Tris pH 8.0, 100 mM KCl, 3 mM 2-mercaptoethanol. Scattering data for the 39 bp duplex DNA substrate was measured at concentrations of 188, 142, and 94 µM by diluting the concentrated stock DNA in 20 mM Tris pH 8.0, 100 mM KCl, 3 mM 2-mercaptoethanol. XRCC1-DNA complex was prepared by mixing XRCC1 and DNA at a 1:1 molar ratio to give a final concentration for each component of 188, 141 and 94 µM. All data were collected on a Rigaku BioSAXS-1000 instrument at 10 °C for 2 hr exposures. SAXSLab 3.0.0r1 software (Rigaku) was used to generate scattering curves. Lack of radiation damage was confirmed by comparing the scattering data at the beginning and end of data collection. Comparison and analysis of the scattering curves was performed using the ATSAS 2.6.0 suite^27^. Samples were devoid of inter-particle interactions as judged from the Guinier plots and the protein judged to be folded from analysis of Kratky plots (Supplementary Fig. S6). Scattering curves used for further processing were generated by merging the low q range from the most diluted samples with the higher q range from the most concentrated samples using the automerge tool in the ATSAS 2.6.0 suite. Radius of gyration and pair-distance distribution functions were determined using Primus and GNOM. The reported molecular weights were calculated based on the volume of correlation^28^.

*Ab initio* modelling of the CDB (XRCC1^219–415^) alone was performed using DAMMIF (Chi square value 1.03). Further rigid body modeling was carried out using BUNCH^27^ with the available XRCC1-BRCT1 structure (PDB ID 2D8M with Chi square value 1.23). *Ab initio* modelling for DNA alone and CDB-DNA complex was performed with DAMMIN (Chi square value 0.99) and MONSA, respectively^27^. MONSA model used scattering intensities of the individual components as input (CDB only, DNA only or CDB-DNA complex) with Chi square values of 3.4, 1.7 and 1.4, respectively. To assess the degree of conformational heterogeneity, we performed ensemble optimization method (EOM)^29^. A randomized pool of 10,000 independent structures were generated using structural information from the XRCC1-BRCT1 atomic model (PDB ID 2D8M). The applied genetic algorithm produced an ensemble of 5 conformational states that best describe the measured SAXS data of the CDB (XRCC1^219–415^) (Chi square of 0.96).

### Live cell imaging and micro-irradiation experiments

HeLa cells were seeded in 35 mm glass petri dishes (250, 000 cells per dish) and transfected 24 hours later with either XRCC1-YFP wild type or P1/3 mutant expression vectors using Lipofectamine 2000 (Life technologies) according to the manufacturers recommendations. Images were acquired as previously reported^30^. Live cell images were captured using a Nikon A1 inverted confocal microscope equipped with an environmental chamber allowing the control of temperature, humidity and gas mixture. 24 hours post-transfection, micro-irradiation was performed with a 405 nm diode laser set to 5% power. Laser power at the fiber exit was 4 mW, and 1 mW at the exit of the 10x objective. Measurements were performed by immobilizing the laser set at 100% in a point bleach with the Digital Handheld Optical Power PM100D from THORLABS. A 488 nm Argon-laser was used to visualize YFP fluorescence. Stimulation and acquisition were performed with the 63x objective at a zoom of 4 using an image size of 512 × 512 pixels. A stimulation line of 5 µm was defined and micro-irradiation performed for 6 sec. Six images were taken before micro-irradiation to quantify a mean fluorescence for basal protein levels within the region and used to normalize further measurements (this value was set to 1). After micro- irradiation, an image was taken every 1 sec over 2 min for short and every 2 min over 62 min for long kinetic experiments. Between 10 and 20 cells were micro-irradiated in each experiment. Fluorescence intensity at the micro-irradiated region was measured for each time point. To quantify the enrichment factor of XRCC1 in the micro-irradiation region the intensity observed for each time point was divided by the mean intensity measured before micro-irradiation. The mean of at least 10 cells is displayed in the graph (Fig. 6). Error bars represent the standard error of mean.

### XRCC1 DNA repair foci analysis

HeLa cells were grown on cover slides and transiently transfected with XRCC1-YFP expression vectors using lipofectamine 2000 (Life technologies) according to the manufacturer’s instructions. 24 hours post transfection, cells were treated with 10 mM H_2_O_2_ for 10 min, washed and incubated for 5 min in Dulbecco modified eagle medium (DMEM) (GIBCO-BRL, Invitrogen). Cells were then fixed with 2% paraformaldehyde for 20 min at room temperature. DNA was stained with 1 µg/mL DAPI (4′, 6′-diamidino-2-phenylindole) for 5 min at room temperature and slides mounted with Dako fluorescence mounting medium. Image acquisition was performed with a Nikon A1 inverted confocal microscope with a 63x objective. Image analysis was performed with ImageJ. The plugins used for foci counting and display were 3D Object Counter^31^ and Volume Viewer respectively.

### Comet assay

CHO EM9 cells (ATCC CRL-1861) were transfected with WT or P1/3 mutant XRCC1-YFP plasmid (described above) using PolyJet (SignaGen) according to manufacturer’s instructions. Stably transfected cells were selected using G418 (1.2–1.6 mg/mL) in AMEM supplemented with 10% FBS, penicillin (100 units/mL, Bioshop), and streptomycin (100 µg/mL) over 3 weeks. Cells expressing fluorescent protein were isolated from a mixed population using automated cell sorting to give samples with >93% of fluorescent cells.

Cells were grown to 80% confluency prior to performing the comet assay. DNA damage was induced by treating cells in growth media (AMEM supplemented with 10% FBS, penicillin, streptomycin) with 10 mM H_2_O_2_ for 10 min at 37 °C before allowing recovery in fresh growth media at 37 °C for 30 min. Cells were then lifted with 1x PBS supplemented with 1 mM EDTA and collected by centrifugation at 100 × g for 3 min. The comet assay was performed using the Comet SCGE assay kit (Enzo) following manufacturer’s instructions. Cells were mixed with 100 µL of LM-Agrose and applied to a slide. All subsequent steps were performed in the dark. The agarose gel was allowed to solidify for 30 min at 4 °C before submerging in lysis solution for 1 h at 4 °C. Slides were then treated with alkaline buffer (200 mM NaOH, 1 mM EDTA) for 1 h at room temperature. Electrophoresis was performed on the slides in alkaline buffer for 30 min (14–16 V, 300 mA) at 4 °C. The slides were then rinsed with water 3 times to remove alkaline buffer and subsequently submersed into 70% ethanol for 5 min. Slides were then air dried at room temperature overnight. To image DNA, slides were covered with Cygreen (2 µg/mL, Enzo) for 15 min and rinsed with water 3 times to remove excess stain. The slides were dried at 37 °C for 15 minutes prior to imaging with an EVOS auto microscope (Life Technologies). Comets were scored using ImageJ and Opencomet software. Data analysis was performed with Microsoft Excel.

## Results

### Identification of a central DNA binding domain within XRCC1

**Figure 1:**
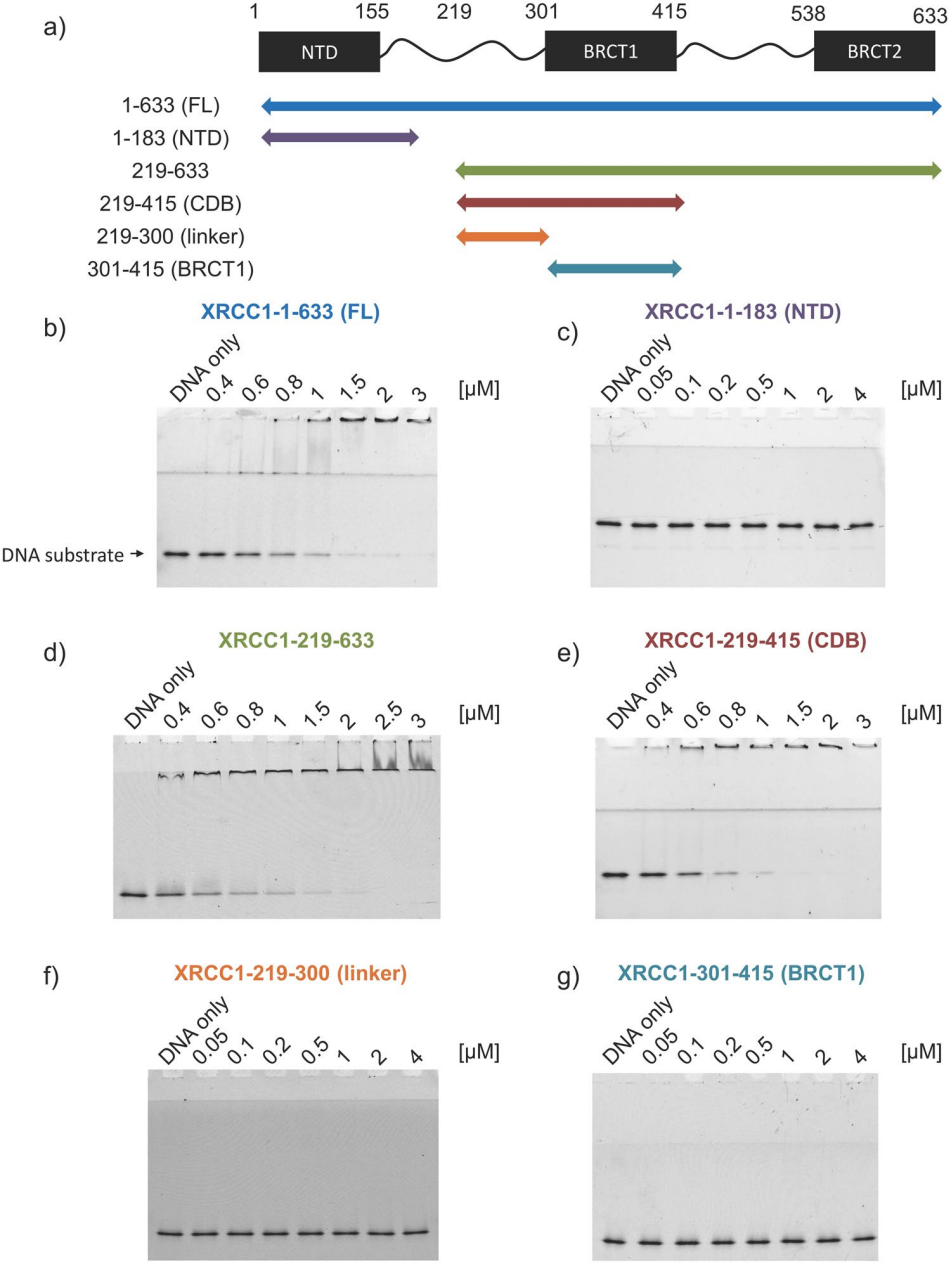
Comparison of DNA binding activities of XRCC1 truncations. (**a**) Domain organization of XRCC1 with truncation boundaries indicated by arrows: Full length (blue), 1–183 (purple), 219–633 (green), 219–415 (red), 219–300 (orange) and 301–415 (cyan). (**b**–**g**) DNA binding activity of XRCC1 truncations (µM concentrations) monitored by electrophoretic mobility shift using fluorescent 39 bp duplex DNA. Discontinuous PAGE was used to facilitate band resolution. Full length gels are in Supplementary Fig. 13.

Although XRCC1 has been shown to bind DNA, the region responsible for this activity has not been well defined. To better characterize the DNA binding function of XRCC1, truncations (Fig. 1a) were generated based on known domain boundaries and stability toward proteolysis^32,33^. The DNA binding capacity of XRCC1 truncations was determined by electrophoretic mobility shift analysis using a 39 bp substrate. The binding affinity was determined by monitoring the disappearance of DNA substrate in the presence of increasing XRCC1 concentrations^34^. Full-length XRCC1 bound DNA with sub-micromolar affinity (0.62 ± 0.01 µM) (Fig. 1b). Although the N-terminal domain (NTD) showed no DNA binding to this substrate (Fig. 1c), binding (0.7 ± 0.01 µM) was observed for the largest proteolysis-resistant domain spanning both BRCT domains (residues 219–633) (Fig. 1d).

To further localize DNA binding activity more extensive truncations were generated. A construct with the C-terminal BRCT2 domain removed (residues 219–415) bound DNA at levels (0.67 ± 0.01 µM) comparable to full-length XRCC1 (Fig. 1e). Consistent with this finding, no DNA binding was observed for the BRCT2 domain alone (Supplementary Fig. S7). Further truncation removing BRCT1 abolished DNA binding (Fig. 1f) suggesting BRCT1 is required for DNA interaction. Surprisingly, when the BRCT1 domain (residues 301–415) was tested on its own, it also failed to bind DNA (Fig. 1g). Since neither the linker region or BRCT1 domain stably bound DNA and DNA binding was only observed when both linker and BRCT1 were present, it suggests that both the linker and BRCT1 domain are required for complete DNA binding activity. Results from this deletion analysis therefore define a central DNA binding domain (CDB, residues 219–415) of XRCC1.

### The CDB domain of XRCC1 lacks DNA substrate specificity

**Figure 2:**
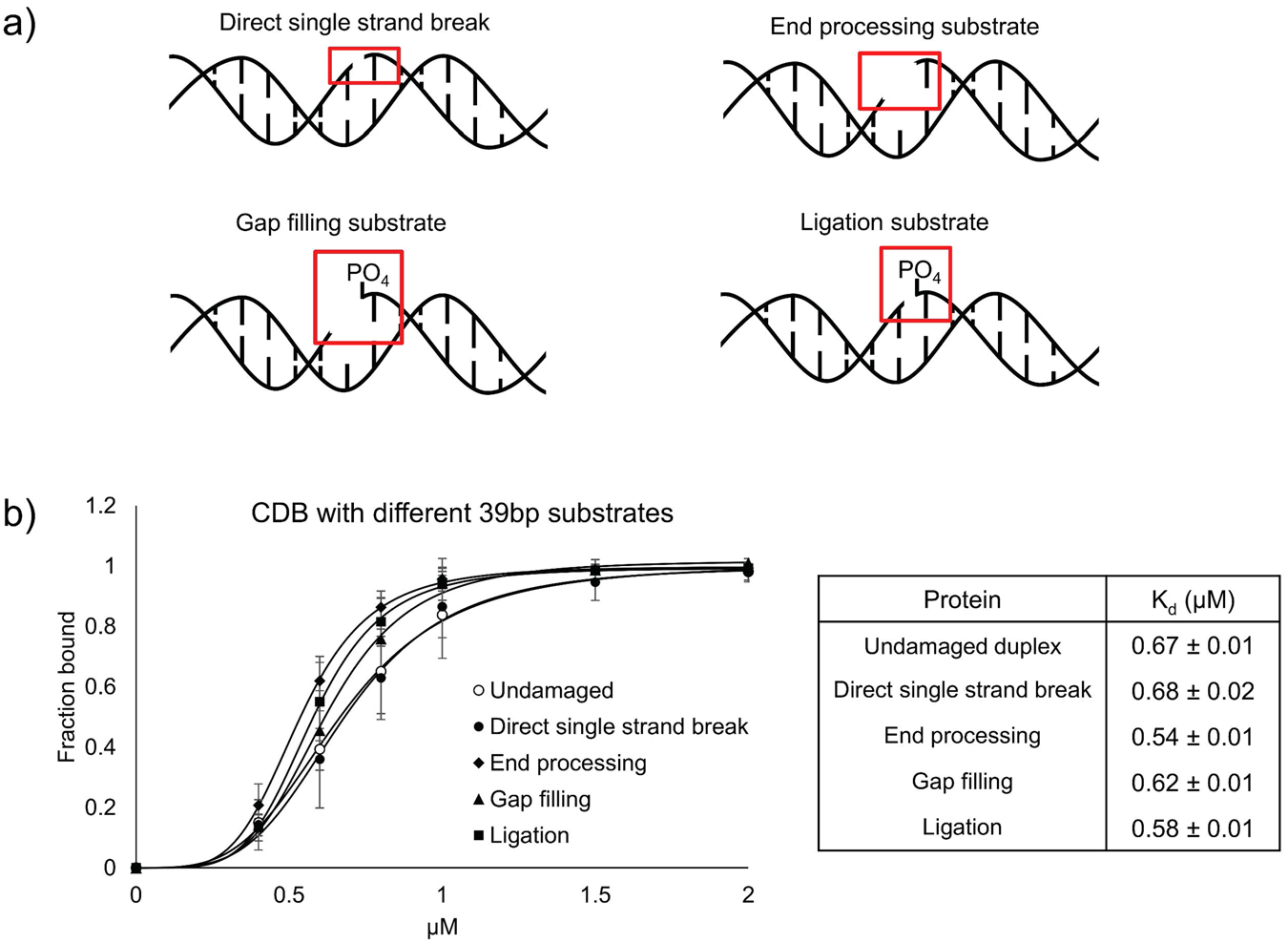
Comparison of XRCC1-CDB binding affinities for different DNA substrates. (**a**) Schematic of DNA substrates analyzed for XRCC1-CDB interaction. All substrates contained a 3′-hydroxyl group at the site of damage. Substrates that contained a 5′-phosphate group at the damage site are labelled. (**b**) Comparison of binding curves of XRCC1-CDB with each substrate showed only minor differences in affinity.

Since the NTD of XRCC1 was previously reported to bind damaged DNA^21^, we next sought to determine if the CDB displayed binding specificity toward DNA with different types of damage (i.e. nicks and gaps). Such binding specificity could be important for XRCC1’s ability to localize and recruit appropriate binding partners to sites of DNA damage at different stages of repair. A series of 39 bp DNA substrates, resembling intermediates observed during repair, were generated (Fig. 2a, Supplementary Fig. S4). These substrates included nicked and gapped DNA with 5′ and 3′OH groups (mimicking a direct single strand break and a substrate requiring end processing, respectively), as well as nicked and gapped DNA with a 5′ PO_4_ and 3′ OH group (resembling substrates ready for ligation and gap filling, respectively). As shown in Fig. 2b, all DNA substrates were bound with comparable affinity, suggesting that the DNA binding activity of XRCC1’s CDB is largely non-specific in nature.

### SAXS analysis of the CDB-DNA complex suggests both BRCT1 and linker regions engage DNA

Although the linker region and BRCT1 domain of XRCC1 were shown to be necessary for DNA binding, it remained unclear how these two portions of the CDB might contribute to form a functional DNA binding unit. To better understand the requirement for both BRCT1 and adjacent N-terminal linker in DNA binding, solution small-angle X-ray scattering (SAXS) data were acquired for the CDB in the absence and presence of DNA.

**Figure 3:**
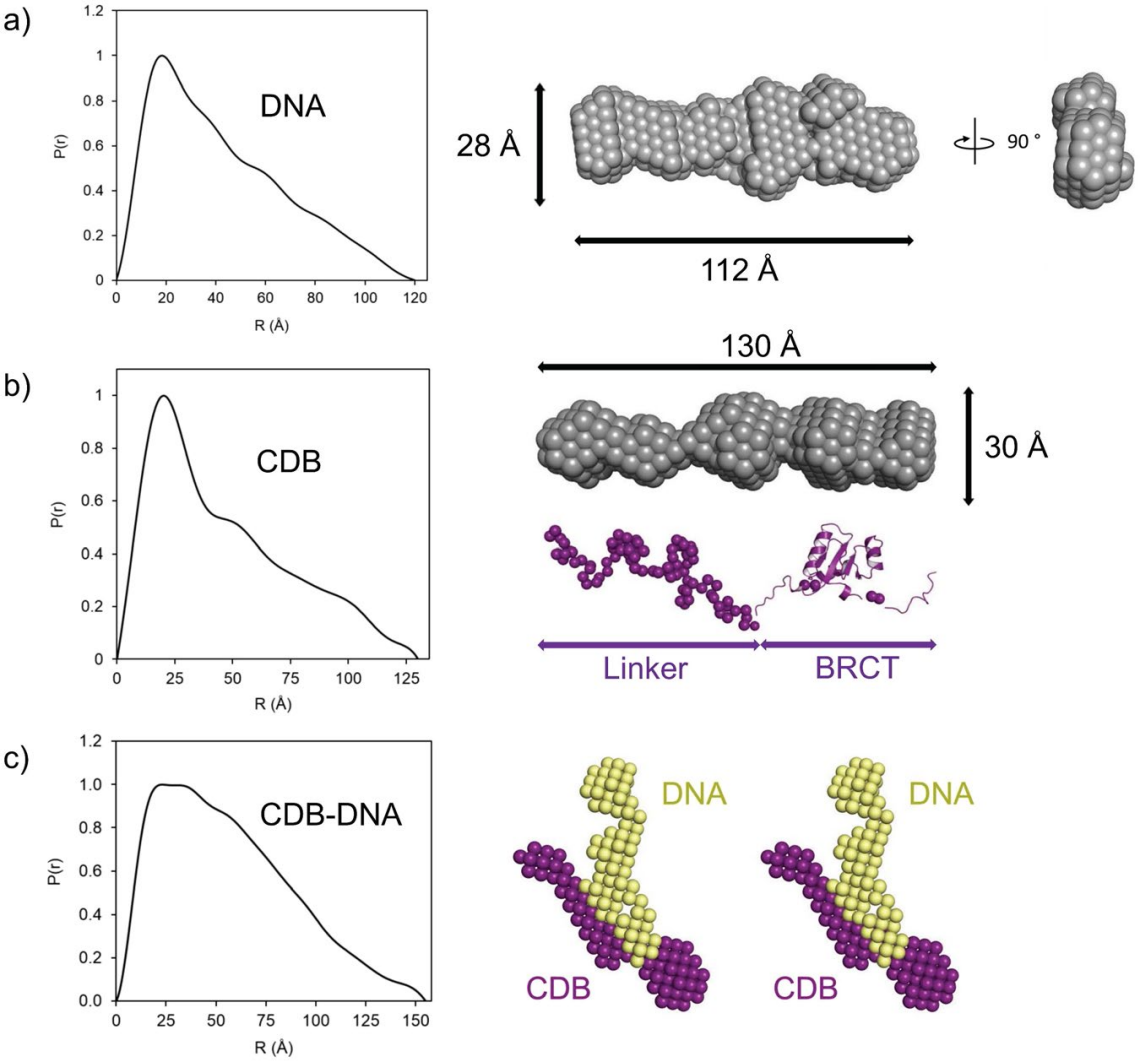
Small angle X-ray scattering of the XRCC1-CDB DNA-bound complex. Pair distribution curves (left) and resulting molecular envelopes (right) of (**a**) 39 bp DNA and (**b**) XRCC1-CDB with the estimated dimensions given in angstroms. An *ab initio* model for XRCC1-CDB was generated with DAMMIF (grey model) with subsequent BUNCH modelling (purple) to populate atoms not present in the BRCT1 domain determined by NMR (PDB 2D8M). (**c**) The pair distribution function for the XRCC1-CDB DNA-bound complex is shown in the left side of the panel. A corresponding MONSA generated model is shown in stereo. Purple spheres correspond to XRCC1-CDB while the yellow spheres correspond to 39 bp DNA.

A*b initio* modeling of the DNA scattering curve confirmed its elongated conformation (Fig. 3a) and resulted in models with dimensions (~28 × 20 × 112) comparable to the size of a 39-bp canonical DNA (20 × 20 × 133 Å)^33^. SAXS analysis of the CDB alone indicated a monomeric state in solution (molecular weight calculated from the volume of correlation) with an elongated structure. Rigid body modeling with a previous crystal structure of the BRCT1 domain (PDB 2D8M) and *ab initio* modelling of the preceding linker, suggested that the CDB region of XRCC1 adopts an extended conformation (Fig. 3b) with approximate dimensions of 30 × 30 × 130 Å. However, further ensemble optimization method (EOM) analysis (Supplementary Fig. S8) indicated that the extended linker region was able to adopt multiple conformations relative to the BRCT domain.

We then collected scattering data for the DNA-bound CDB complex at a 1:1 binding stoichiometry (Supplementary Table S1). An *ab initio* model of the complex was generated using the multi-phase *ab initio* modeling program MONSA, where scattering data of the complex (Fig. 3c) was interpreted using scattering data from individual components (CDB and/or DNA alone). The resulting chi-square values for models fit with scattering intensities of DNA alone, CDB alone and complex were 1.7, 3.4 and 1.4, respectively; indicating that DNA within the complex remains unaltered from its unbound state (Fig. 3a), and that XRCC1 does not induce significant changes in DNA geometry upon binding. The fact that chi-square values for model fitting were significantly higher for the CDB further suggests that complex formation may result in some amount of conformational change within the CDB. The CDB was observed to interact with DNA predominantly through the linker region and to a lesser extent BRCT1 (Fig. 3c). Taken together, these results further support findings from deletion studies suggesting that the CDB of XRCC1 interacts with DNA through both the extended linker and the BRCT1 domain.

### Identification of key DNA binding residues within the CDB of XRCC1

**Figure 4:**
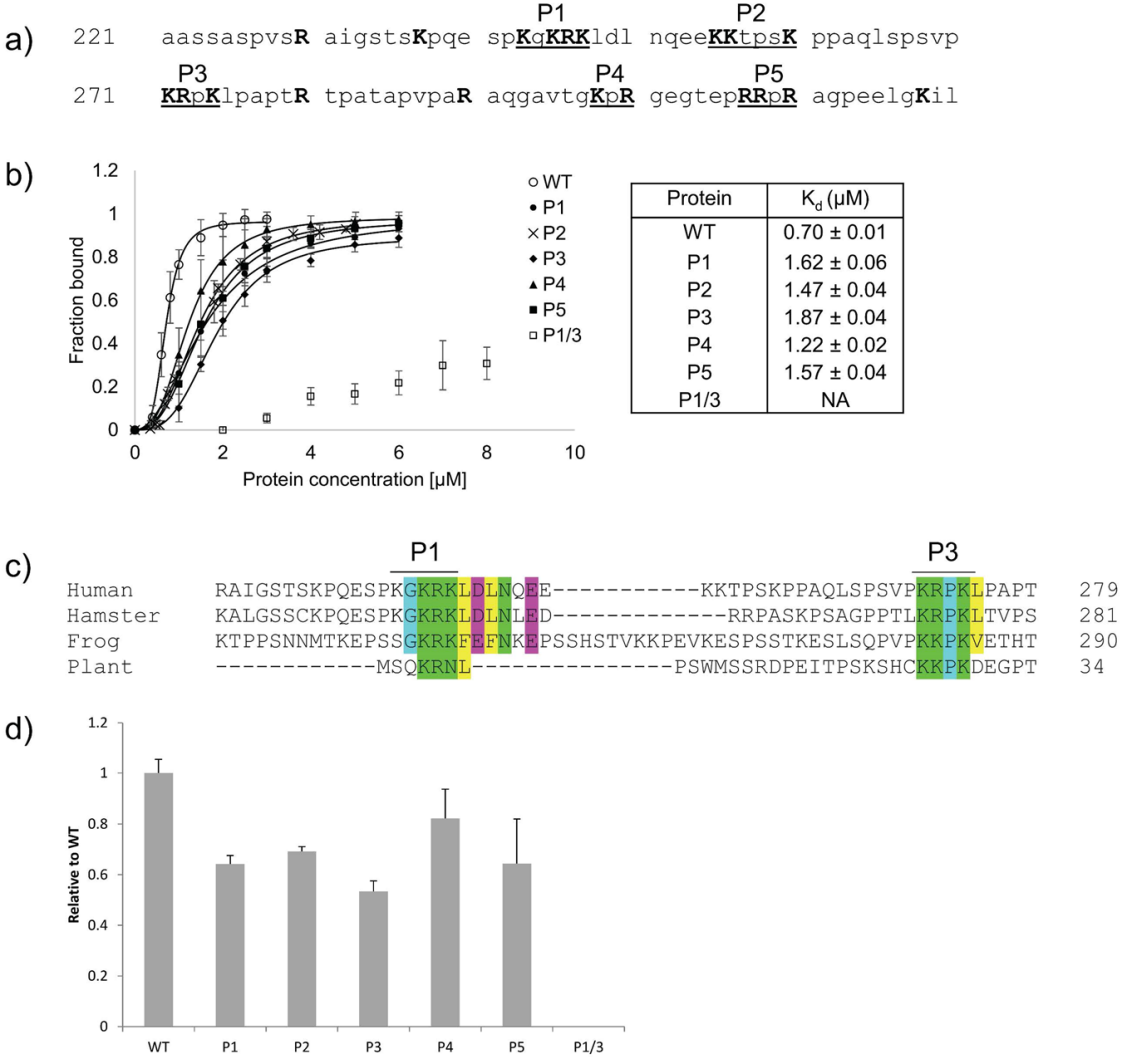
Comparison of DNA Binding affinity for XRCC1-CDB mutants. (**a**) Sequence of the XRCC1 N-terminal linker. Positively charged residues that were targeted for alanine substitution are highlighted in bold. (**b**) Binding curves generated from EMSA analysis for each alanine substituted mutant (right) and the corresponding K_d_ values (left). (**c**) Sequence alignment of XRCC1 from human, hamster, frog and Arabidopsis (plant). Conserved positively charged residues, green; glycine and proline, cyan; negatively charged residues, pink; and hydrophobic residues, yellow. (**d**) A comparison of DNA binding levels for mutant and wild type XRCC1 at 2 µM protein concentration. Each experiment was repeated three times. Mutant P1/3 had no measurable DNA binding activity at this concentration.

To determine if DNA binding of XRCC1 is important for recruitment and/or retention at sites of DNA damage, we first needed to identify key residues responsible for mediating DNA binding. Based on SAXS analysis, the most extensive DNA binding interface within the CDB of XRCC1 appeared to be located within the linker region. This and the fact that BRCT1 is known to facilitate other interactions (ie. with PARP1) lead us to specifically target the CDB linker region to locate residues critical for mediating DNA binding. Examination of the amino acid sequence within the CDB revealed several positively charged residues (Fig. 4a) located in 5 clusters (designated P1–P5). Charged residues within each cluster were changed to alanine and the DNA binding capacity of each mutant determined (Fig. 4b). Although none of the individual mutants (P1 – P5) significantly diminished DNA binding, most exhibited a modest reduction in affinity. We reasoned that since residues within P1 and P3 are highly conserved (Fig. 4c) they might contribute more to DNA binding. Indeed, combining P1 and P3 amino acid changes into a single mutant (P1/3) dramatically reduced levels of DNA binding (Fig. 4b). At a fixed protein concentration (2 µM) where WT protein is fully bound, the P1/3 mutant showed almost no detectable DNA binding (Fig. 4d). With the current data it is not possible to say with certainty whether the linker region or BRCT1 domain contribute more to DNA binding affinity. Nevertheless, these findings suggest that DNA binding activity of XRCC1 can be disrupted by mutation at two positively charged clusters (P1 and P3) in the CDB linker without altering residues within the BRCT1 domain.

### XRCC1 P1/3 mutation causes early release from sites of DNA damage

To evaluate the impact of reduced XRCC1 DNA binding on its cellular function, we compared the localization of wild type and P1/3 mutant XRCC1 in HeLa cells in response to DNA damage. Yellow fluorescent protein (YFP) was fused to the C-terminus of each protein to permit localization of XRCC1 using live cell imaging. Since residues altered within the P1/3 mutant overlap with the bipartite NLS sequence of XRCC1^35^, a nuclear localization signal (DPKKKRKV) was added to the C-terminal YFP fusion for both wild type and mutant XRCC1. Addition of the YFP-NLS fusion did not significantly alter DNA binding (Supplementary Fig. S9).

**Figure 5:**
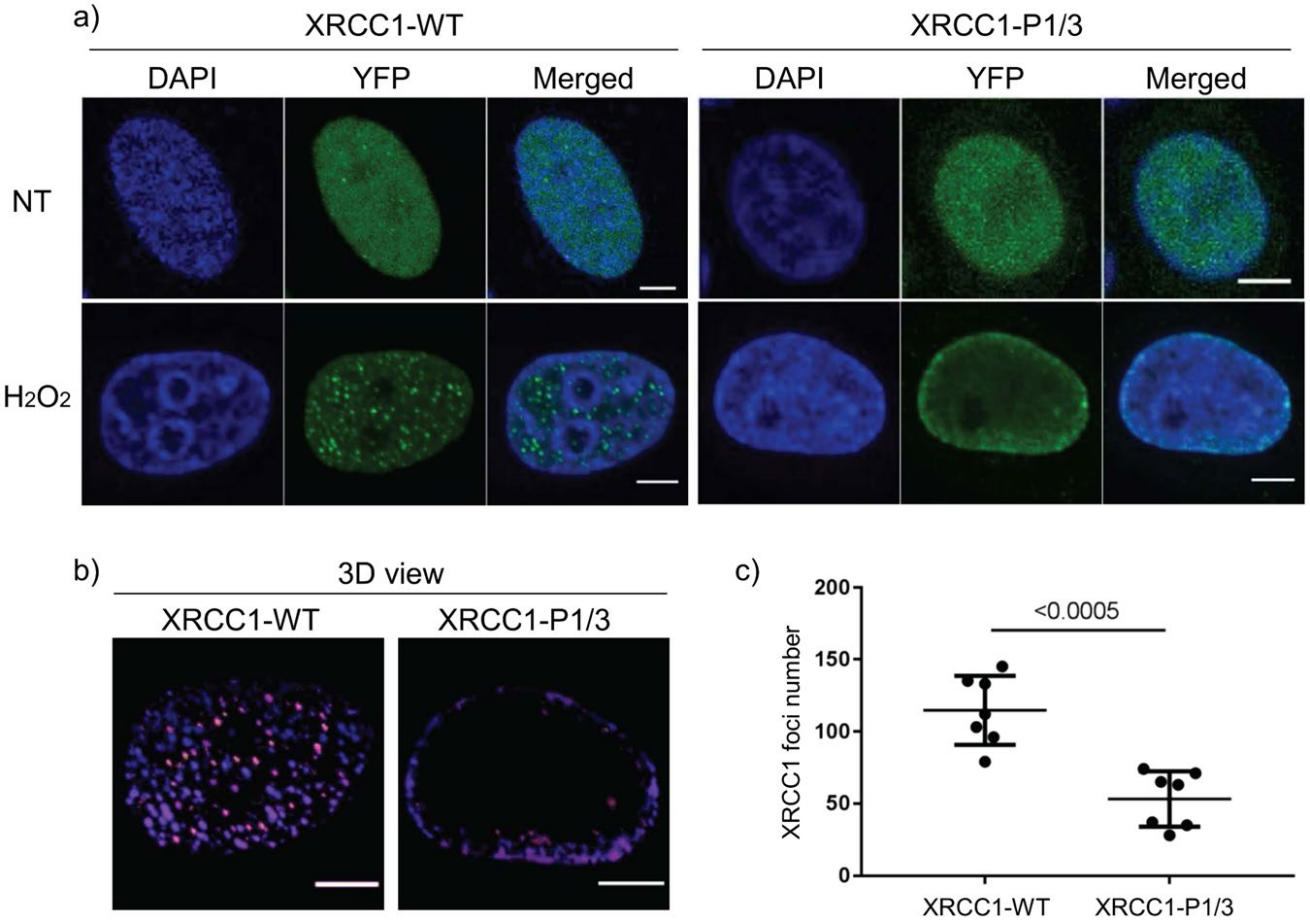
Comparison of XRCC1 foci formation following 10 mM H2O2 treatment. (**a**) Cells expressing wild type XRCC1 (left) or P1/3 variant (right). DNA, stained with DAPI (blue colour); XRCC1 fused with YFP (green). One single confocal image taken at the center of the nucleus is presented. (**b**) 3D-stacks were acquired and visualized with Volume Viewer. The colour code reflects the position of the foci in the 3D space. (**c**) The foci number were counted with the ImageJ plugin 3D Object Counter. Results for 7 representative cells expressing each of the XRCC1 variants are displayed in the scatter dot plot. Graph representation and Mann-Whitney statistical test were performed with GraphPad Prism version 7, 02. Scale bars, 5 µm.

The recruitment of XRCC1 to sites of single-strand DNA breaks following exposure to hydrogen peroxide (H_2_O_2_) has been well documented. WT XRCC1 rapidly localizes to distinct repair foci following DNA damage^36,37^. To test the capacity of the P1/3 mutant to be recruited to repair foci, cells expressing wild type or mutant (P1/3) XRCC1 fused to YFP were treated with 10 mM H_2_O_2_ and monitored for foci formation. As previously shown, wild type XRCC1 incorporated into foci following exposure to H_2_O_2_ (Fig. 5). In contrast, the P1/3 mutant showed a sharp reduction in the number and prominence of foci observed (Fig. 5). This finding suggests that DNA binding of the CDB plays a role in XRCC1 foci formation, potentially by altering recruitment and/or retention of XRCC1 at sites of damage.

**Figure 6:**
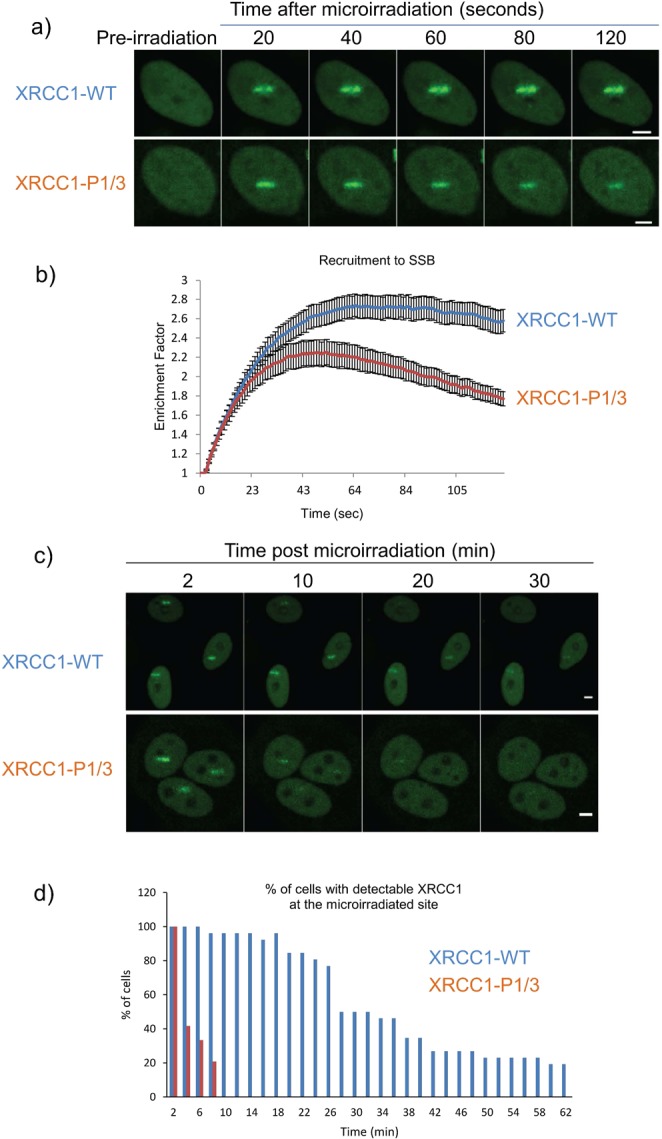
Recruitment of XRCC1 and XRCC1-P1/3 mutant to sites of micro-irradiation damage. (**a**) Time course of XRCC1 DNA damage localization 20–120 seconds post-irradiation. (**b**) Relative levels of XRCC1 and XRCC1-P1/3 mutant localization over 120 seconds post-irradiation. (**c**) Time course of XRCC1 DNA damage localization 2–30 min post-irradiation. (**d**) Comparison of number of cells showing detectable localization of XRCC1 at damage sites over 62 min following irradiation. Blue and orange bars represent wild type and P1/3 mutant XRCC1, respectively. Scale bars, 5 µm.

To distinguish between these possibilities, we analyzed XRCC1 recruitment and retention at sites of DNA damage in cells micro-irradiated to generate localized DNA single strand breaks. While both wild type and P1/3 proteins were initially recruited to damage sites at similar rates (Fig. 6a,b; 0–10 seconds post irradiation), the P1/3 variant failed to achieve the same extent of accumulation (~85% of wild type). Moreover, wild type protein remained at damage sites for extended times (between 40–120 seconds) compared to P1/3 mutant, which decreased steadily during the same period and by 120 seconds had dropped to ~50% of wild type (Fig. 6a,b). This effect became more pronounced over time with wild type protein still detected at sites of damage after 60 min, while P1/3 was no longer present after 10 min (Fig. 6c,d).

Taken together, the findings from both peroxide treatment and micro-irradiation studies suggest that while diminishing DNA binding activity within the CDB of XRCC1 does not appreciably alter initial recruitment to sites of DNA damage, retention at repair foci is significantly reduced.

### Reduction in XRCC1 DNA binding increases rate of DNA repair

**Figure 7:**
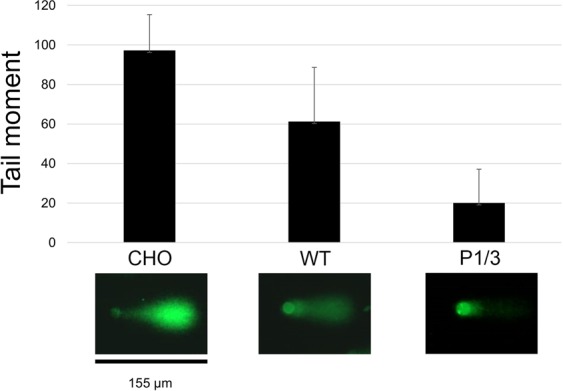
Effect of P1/3 mutation on DNA repair of single strand breaks. Relative repair efficiency of single strand breaks compared for CHO cells containing an XRCC1 knock out and the same cells complemented with either WT or P1/3 mutant XRCC1. The average tail moments are shown for each cell line after 30 min of recovery following initial damage with 10 mM hydrogen peroxide. Representative images from comet assays, which were used for average tail moment calculation, are provided in the lower panels.

Given the significant effect that altering XRCC1 DNA binding affinity had on retention at damage foci, we next wanted to determine if reduced DNA binding might impact repair efficiency. XRCC1 deficient cells (EM9) were transfected with WT or P1/3 mutant XRCC1, challenged with hydrogen peroxide and monitored for repair using the comet assay. As shown in Fig. 7, tail moments were most pronounced when XRCC1 was absent, indicating a defect in repair ability. Interestingly, the P1/3 mutant with reduced DNA binding restored DNA duplex integrity more efficiently relative to WT XRCC1.

## Discussion

The repair of modified DNA bases and single strand breaks requires well-coordinated events orchestrated by the scaffolding protein XRCC1 [reviewed in^1,8^]. In order to effectively recruit and displace binding proteins that act at various stages during repair, XRCC1 remains closely associated with the site of DNA damage throughout the repair process. XRCC1 has been shown to bind DNA *in vitro*^20–23^; however, it is not clear where DNA binding is localized within XRCC1, and if recruitment, retention and/or repair is dependent on XRCC1’s direct interaction with DNA. We therefore sought to identify minimal DNA binding determinants within XRCC1 in order to evaluate their impact on recruitment and retention to sites of DNA damage as well as corresponding effects on repair.

### Mechanism of XRCC1 non-specific DNA binding

Results presented here localize the non-specific DNA binding activity of XRCC1 to a region spanning residues 219–415 (Fig. 1). This CDB domain contains multiple determinants for DNA binding, including the N-terminal linker region and BRCT1 domain. SAXS analysis of the CDB revealed an elongated monomer in solution. Although it is not possible to say with certainty where the linker and BRCT domain are precisely located within the calculated SAXS envelopes, analysis using DAMMIF and BUNCH suggest that the BRCT domain is located on one end of the elongated envelop with the linker region extending toward the opposing end. When bound to DNA, the CDB appeared to undergo a conformational change as judged by elevated Chi-squared values. Such a conformational change could serve as a means of transitioning between different binding partners (eg. PARP1 vs DNA). In contrast to the CDB, DNA appeared to remain unaltered in its geometry when in complex. SAXS models further suggest that the CDB engages DNA through interaction with both the linker region and BRCT1 domain. These findings are consistent with binding studies showing a dependence of both the linker and BRCT1 for DNA complex formation. Whether the contribution of the BRCT domain is dependent on direct contact with DNA or indirect scaffolding function to allow the linker to properly bind DNA remains unclear. Given the requirement of BRCT1 for DNA binding, and the well-known role of BRCT1 for interacting with binding partners such as PARP1, it is tempting to speculate that binding of XRCC1 to DNA might influence the interaction with other binding partners, and perhaps the converse.

Results from our DNA binding studies (Fig. 2) indicate that the CDB shares similar affinity for a variety of different types of DNA, suggesting a non-specific mode of interaction. Similar binding was also observed with pUC19 DNA in linear, nicked and supercoiled forms suggesting no requirement for DNA ends (Supplementary Fig. S10). SAXS analysis of the CDB demonstrated no obvious preference for DNA ends or altered DNA geometry implying that, from a structural perspective, the CDB has limited requirements for binding DNA. Nevertheless, the affinity of the CDB for DNA is quite high (~700 nM) and has a preference for DNA that is at least 35 bp (Supplementary Fig. 11). As one would expect for a non-specific DNA binding protein that contacts negatively charged phosphates within DNA, mutation of several positively charged residues were required to fully ablate DNA binding (Fig. 4). Taken together, the finding that the CDB of XRCC1 exhibits no obvious structure or sequence specificity for DNA binding suggests that XRCC1 would not likely use this activity for its initial recruitment to sites of DNA damage.

### DNA binding within the CDB of XRCC1 is dispensable for recruitment to sites of DNA damage

In cells exposed to micro-irradiation-induced DNA damage, XRCC1 recruitment was observed even when DNA binding within the CDB was compromised (Fig. 6). It should be noted, however, that even though DNA binding of the P1/3 mutant was reduced by at least an order of magnitude, residual affinity for DNA may still contribute to the stability of the repair complex during recruitment. Nevertheless, the finding that DNA binding activity within the CDB appears to be dispensable for XRCC1’s initial recruitment to damage sites is consistent with the current understanding that XRCC1 is recruited following SSBR/BER initiation by PARP1 or DNA glycosylases^11–13,36–38^. These repair factors bind damage sites with high affinity and specificity, and therefore XRCC1 may not require an independent damage sensing function to be recruited to repair sites. In support of this suggestion, XRCC1 fails to localize to sites of peroxide damage when PARP ADP-ribosylation activity is ablated^39^. Given that XRCC1’s BRCT1 domain is required for interaction with PARP1^11^, an interesting possibility arises that residues involved in DNA interaction may overlap with PARP1 binding. However, this does not seem likely since the P1/3 mutant was recruited to damage sites and recruitment has been shown to be dependent on PARP1 interaction. Thus, it would appear that disruption of DNA binding in the P1/3 mutant does not impact interaction of XRCC1 with PARP1.

Although our findings suggest that XRCC1 is recruited to damage sites by interaction with repair factors and not DNA directly, prior studies have identified a DNA damage sensing activity within the N-terminal domain (NTD)^21^. Therefore, it remains possible that XRCC1 may use damage sensing within the NTD to aid in its initial recruitment to repair sites; however, we and others have been unable to reproduce damage sensing activity with purified protein *in vitro*^39^ (and Supplementary Fig. S12). Furthermore, binding of the NTD to POLβ (and not DNA) has been shown to be biologically important for repair^40,41^. The significance of the NTD interaction with DNA therefore remains unclear.

The finding that DNA binding of XRCC1 can be localized to the CDB, and that diminishing this activity does not affect initial recruitment to sites of DNA damage is consistent with a model for XRCC1 recruitment involving interaction with binding partners and not DNA per se.

### XRCC1 retention at sites of DNA damage is dependent on DNA binding and regulates repair efficiency

Although DNA binding activity within the CDB of XRCC1 appears to be dispensable for its initial recruitment to DNA damage sites, the finding that disruption of DNA binding greatly increases the rate of XRCC1 dissociation from damage sites (Fig. 6) suggests DNA binding is required for its retention at repair foci. It is currently unclear how the CDB mediates retention of XRCC1 at damage sites. It is possible that XRCC1 may be initially recruited to damage sites primarily through interaction with early responding repair factors such as PARP-1 and only makes significant use of its DNA binding activity to stabilize the SSBR complex when recruitment factors such as PARP-1 are no longer present. Consistent with this idea, a comparison of retention times among repair factors suggests that XRCC1 remains at DNA repair sites considerably longer than early response factors such as PARP1^42^. It is also possible that XRCC1 DNA binding and retention may be enhanced by XRCC1 binding partners that participate at later stages in repair (i.e. Polβ, APTX and DNA Lig3a). Indeed, abolishing interaction with Lig3a results in decreased retention of XRCC1 at DNA damage sites^43^. Since mutations that disrupt DNA binding do not localize to regions of XRCC1 that mediate interaction with binding partners it is unlikely that diminished retention at DNA damage sites is the result of loss of XRCC1 association with binding partners. Rather, the early release of mutant XRCC1 from sites of DNA damage suggests that partner proteins cannot compensate for the lack of CDB-DNA interaction, and that the CDB-DNA interaction is key to XRCC1 stabilization and retention at damaged sites.

Given the significant reduction in retention of XRCC1 at damage foci and the reliance of XRCC1 to recruit key repair factors such as POLβ, PNKP, Lig3a^43^, it was surprising that a defect in XRCC1 DNA binding enhanced repair efficiency (Fig. 7). The data suggest that rapid repair is dependent on initial recruitment of XRCC1 since the absence of XRCC1 (EM9 negative control) resulted in reduced repair. It is not clear, however, why a DNA binding mutant that diminishes retention at damage sites would enhance repair of single strand breaks. Possible reasons for this observation may reflect a requirement of WT XRCC1 to be retained for longer periods in order to help ensure repair accuracy or perhaps limit access by other repair factors (ie. those involved in long-patch BER)^44^. Nevertheless, the data presented here suggest an important role of the CDB for XRCC1 retention at damage foci and regulation of repair efficiency.

## Conclusion

DNA single stranded break and base excision repair require the scaffolding protein XRCC1. While XRCC1 is known to interact with a multitude of protein partners, its interaction with DNA has remained less well characterized. Furthermore, the consequence of XRCC1-DNA binding, or lack thereof, has not been observed inside cells. Our findings now suggest that within XRCC1, a non-specific DNA binding region comprised of the N-terminal linker and central BRCT domain (termed the CDB) is required for retention to sites of DNA damage and that this interaction plays a key role in regulating the rate of repair, perhaps by limiting access of other repair factors involved in backup repair pathways.

## Supplementary information

Supplementary Information

## Data Availability

The datasets generated during and/or analysed during the current study are available from the corresponding author on reasonable request.
