# Supplementary material for: Identification of an XRCC1 DNA binding activity essential for retention at sites of DNA damage
Source: Sci Rep. 2019 Feb 28;9:3095. doi: 10.1038/s41598-019-39543-1 (PMC6395731; doi:10.1038/s41598-019-39543-1)
Supplement: Supplementary file 1 — Supplementary Information [file 41598_2019_39543_MOESM1_ESM.pdf]

Identification of an XRCC1 DNA binding activity essential for retention at sites of DNA damage

Mac C.Y. Mok[a], Anna Campalans[b], Monica C. Pillon[a], Alba Guarné[a], J. Pablo Radicella[b], Murray S. Junop* [a, c]

[a] Department of Biochemistry and Biomedical Sciences, McMaster University, Hamilton, Ontario, L8S4K1, Canada

[b] Institute of Cellular and Molecular Radiobiology, CEA, UMR967 INSERM, F-92265 Fontenay aux Roses, France

[c] Department of Biochemistry, Schulich School of Medicine and Dentistry, Western University, London, Ontario, N6A5C1, Canada

XRCC1-Forward-1 (Gateway)
GGGGACAAGTTTGTACAAAAAAGCAGGCTTAGATTACGATATCCCAACGACCGAAAACCTGTATTTTCAGGGCATGCCGGAGATCCGCCTCC

XRCC1-Forward-219 (Gateway)
GGGGACAAGTTTGTACAAAAAAGCAGGCTTAGATTACGATATCCCAACGACCGAAAACCTGTATTTTCAGGGCTCTAGTGCTGCCTCCTCAGCC

XRCC1-Forward-301 (Gateway)
GGGGACAAGTTTGTACAAAAAAGCAGGCTTAGATTACGATATCCCAACGACCGAAAACCTGTATTTTCAGGGCGGAGAAGGCACCGAGCCCAG

XRCC1-Forward-521 (Gateway)
GGGGACAAGTTTGTACAAAAAAGCAGGCTTAGATTACGATATCCCAACGACCGAAAACCTGTATTTTCAGGGCGAGAACACGGACAGTGAGGAA

XRCC1-Reverse-633 (Gateway)
GGGGACCACTTTGTACAAGAAAGCTGGGTCTCAATGGTGATGGTGATGGTGGGCCTGCGGCACCACCCC

XRCC1-Reverse-183 (Gateway)
GGGGACCACTTTGTACAAGAAAGCTGGGTCTCAGTTGGCGCTCTCATCCTC

XRCC1-Reverse-415 (Gateway)
GGGGACCACTTTGTACAAGAAAGCTGGGTCTCAGGCCTCATCCTCCTCACTG

XRCC1-Reverse-300 (Gateway)
GGGGACCACTTTGTACAAGAAAGCTGGGTCTCATCGGGGTTTGCCTGTCACTGC

XRCC1-Reverse-NLS fusion (Gateway)
GGGGACCACTTTGTACAAGAAAGCTGGGTCTTATACCTTTCTCTTCTTTTTTGG

XRCC1-Forward-219 (LIC)
ATTTCCAGGGAGCAGCCGCATCTAGTGCTGCCTCCTCAGCC

XRCC1-Reverse-633 (LIC)
CCTGCAAAGCACCGGCCTCAGGCCTGCGGCACCACCCC

Figure S1. Primers used to generate XRCC1 constructs. Each primer is oriented from 5’ to 3’. The primers are labelled forward or reverse with the corresponding number reflecting the first or last amino acid of XRCC1, respectively. Parentheses indicate cloning method used. Different combinations of forward and reverse primers were used to generate desired sizes of XRCC1 open reading frame.

NLS addition
Forward- GATCCAAAAAAGAAGAGAAAGGTATGAGGCCGGTGCTTTGCAGGATC
Reverse- TACCTTTCTCTTCTTTTTTGGATCGGCCTGCGGCACCACCCCA

P1
Forward- GCAGGGGCGGCGGCGTTGGATTTGAACCAAGAAGAAAAGAAGACCC
Reverse- CGCCGCCGCCCCTGCGGGAGACTCCTGGGGCTTGGAG

P2
Forward- GCGGCGACCCCCAGCGCACCACCAGCCCAGCTGTCGCCATCTG
Reverse- TGCGCTGGGGGTCGCCGCTTCTTCTTGGTTCAAATCCAACTTCCTCTTCCCTTT

P3
Forward- GCGGCACCTGCATTGCCAGCTCCAACTCGTACC
Reverse- CAATGCAGGTGCCGCGGGAACAGATGGCGACAG

P4
Forward- GCACCCGCAGGAGAAGGCACCGAGCC
Reverse- TCCTGCGGGTGCGCCTGTCACTGCCC

P5
Forward- GCAGCACCCGCAGCTGGCCCAGAGGAGCTGGG
Reverse- AGCTGCGGGTGCTGCGGGCTCGGTGCCTTCTCC

Figure S2. Primers used for site directed mutagenesis and NLS insertion to generate XRCC1 mutants/fusions. Each primer is oriented from 5’ to 3’. The underlined region is the mutation-containing overhang and the remaining sequence corresponds to plasmid-overlapping region.

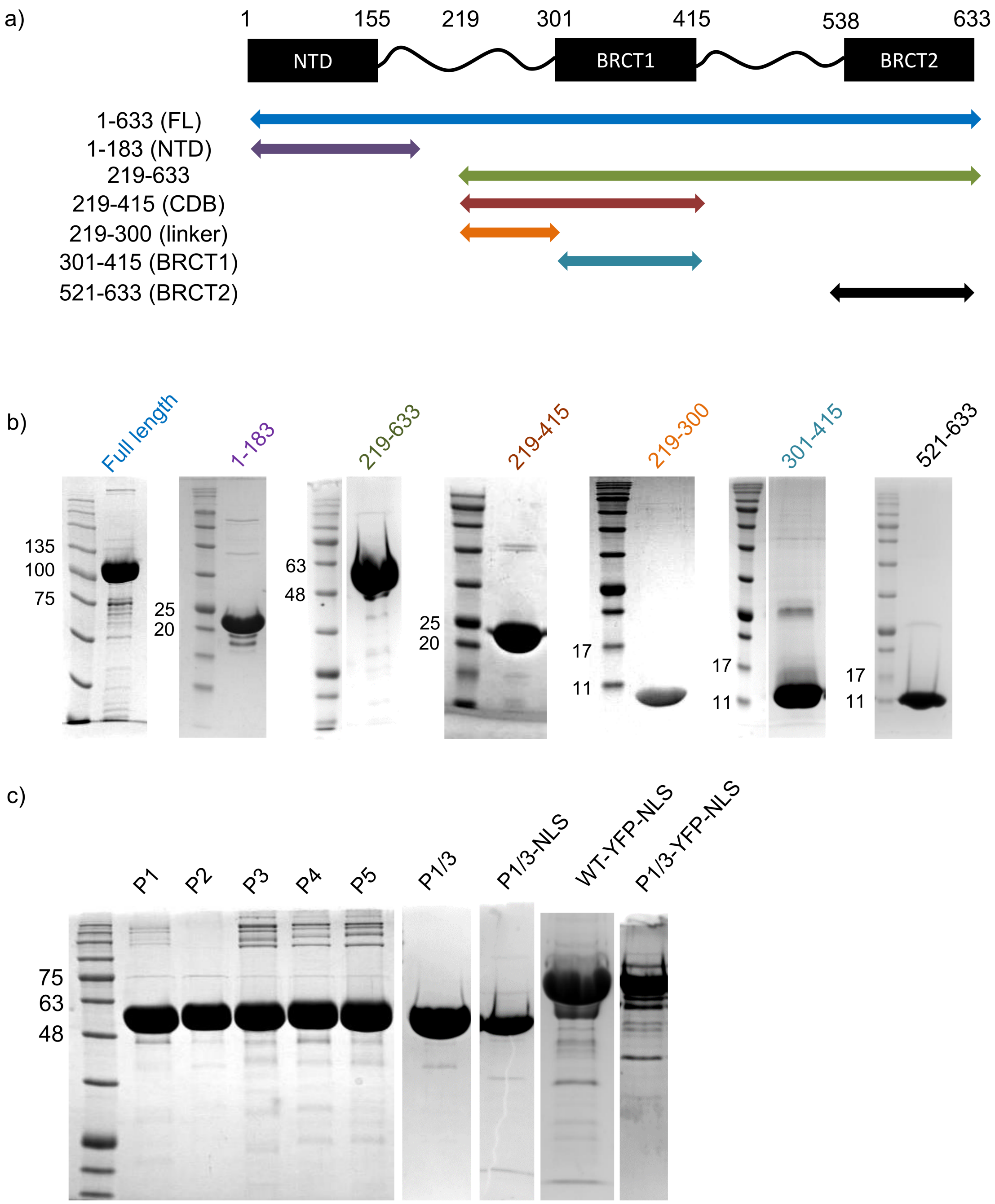

Figure S3. Purified XRCC1 mutants and truncations. A) Domain organization of XRCC1 with domain boundaries indicated by numbers. XRCC1 truncations used in this study are indicated by horizontal colored arrows. **B)** SDS-PAGE gels illustrating purity of XRCC1 constructs used in DNA binding experiments: full length (70k Da), 219-633 (46k Da), 1-183 (20k Da), 219-415 (21k Da), 301-415 (13k Da), 219-300 (8k Da). **C)** SDS-PAGE gel showing purified XRCC1$^{219\text{-}633}$ mutants. All mutants and XRCC1-P1/3-NLS are approximately 46kDa, but migrate with an apparent molecular weight of approximately 55k Da. The XRCC1-YFP-NLS fusions are approximately 75k Da. The total protein loaded ranged between 25-100 µg. A molecular weight marker is shown on the left (kDa).

Duplex undamaged substrate:

5'CGAGCCATGGCCGC**TA**GGCAGATTTTTTGCGGTGCCAGG

3′GCTCGGTACCGGCGATCCGTCTAAAAAACGCCACGGTCC

End processing substrate:

5'CGAGCCATGGCCGCT_GGCAGATTTTTTGCGGTGCCAGG

3′GCTCGGTACCGGCGATCCGTCTAAAAAACGCCACGGTCC

Gap filling substrate:

PO^4

5'CGAGCCATGGCCGCT_^GGCAGATTTTTTGCGGTGCCAGG

3′GCTCGGTACCGGCGATCCGTCTAAAAAACGCCACGGTCC

Direct single strand break substrate:

5'CGAGCCATGGCCGC**T^A**GGCAGATTTTTTGCGGTGCCAGG

3′GCTCGGTACCGGCGATCCGTCTAAAAAACGCCACGGTCC

Ligation substrate:

PO^4

5'CGAGCCATGGCCGC**T^A**GGCAGATTTTTTGCGGTGCCAGG

3′GCTCGGTACCGGCGATCCGTCTAAAAAACGCCACGGTCC

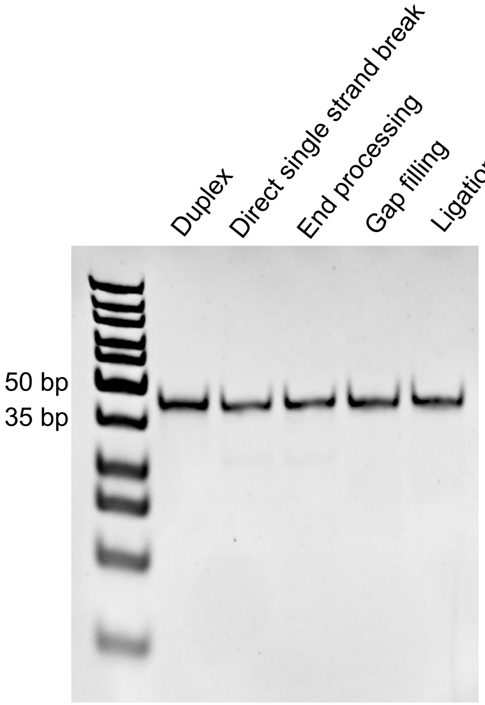

Figure S4. DNA substrates used in DNA binding studies. DNA substrates were chosen to resemble different repair intermediates. The duplex DNA substrate is 39bp long without any modifications. The End processing substrate is missing a base which is indicated by an underscore, and contains a 3' hydroxyl group on the thymine and 5' hydroxyl group on the guanine. The gap filling substrate contains a missing base which is indicated by an underscore as well as a 3' hydroxyl group on the thymine and a 5' phosphate group on the guanine. The direct single strand break substrate contains a nick which is indicated by an accent mark and also contains a 3' hydroxyl group on the thymine and a 5' hydroxyl group on the adenine. The ligation substrate contains a nick which is indicated by an accent mark and also contains a 3' hydroxyl group on the thymine and 5' phosphate group on the adenine. A 3' 6-FAM fluorescent label was added to the bottom strand of each substrate. Purified annealed DNA substrates (1 ng) used in binding studies run on 15% 0.5 TBE gel and stained with ethidium bromide. A DNA ladder (in base pairs) in shown in the left most lane for reference.

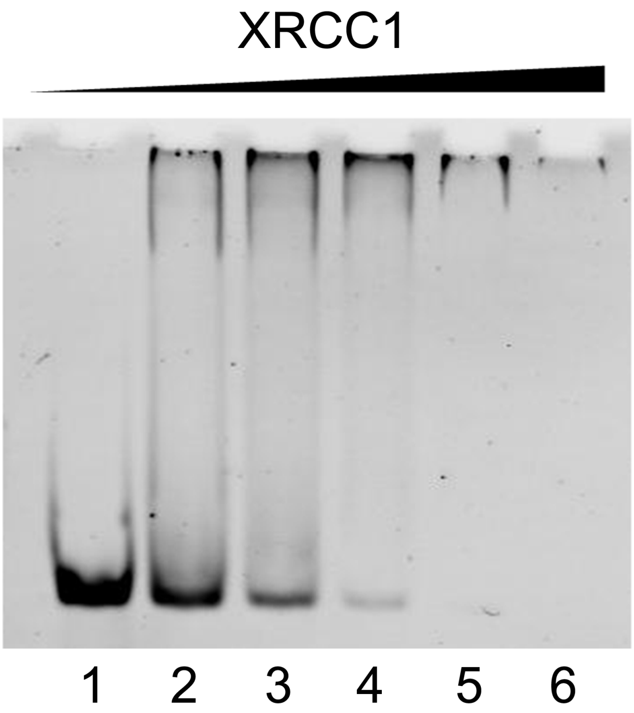

Figure S5. EMSA analysis of XRCC1 DNA binding with buffer used for SAXS studies. 39 bp duplex was incubated with increasing amounts of XRCC1 (lane 1, no protein; lane 2 – 6: 1, 2, 4 ,8 16 uM XRCC1). DNA substrates used in DNA binding studies. Estimated Kd is approximately 1 uM.

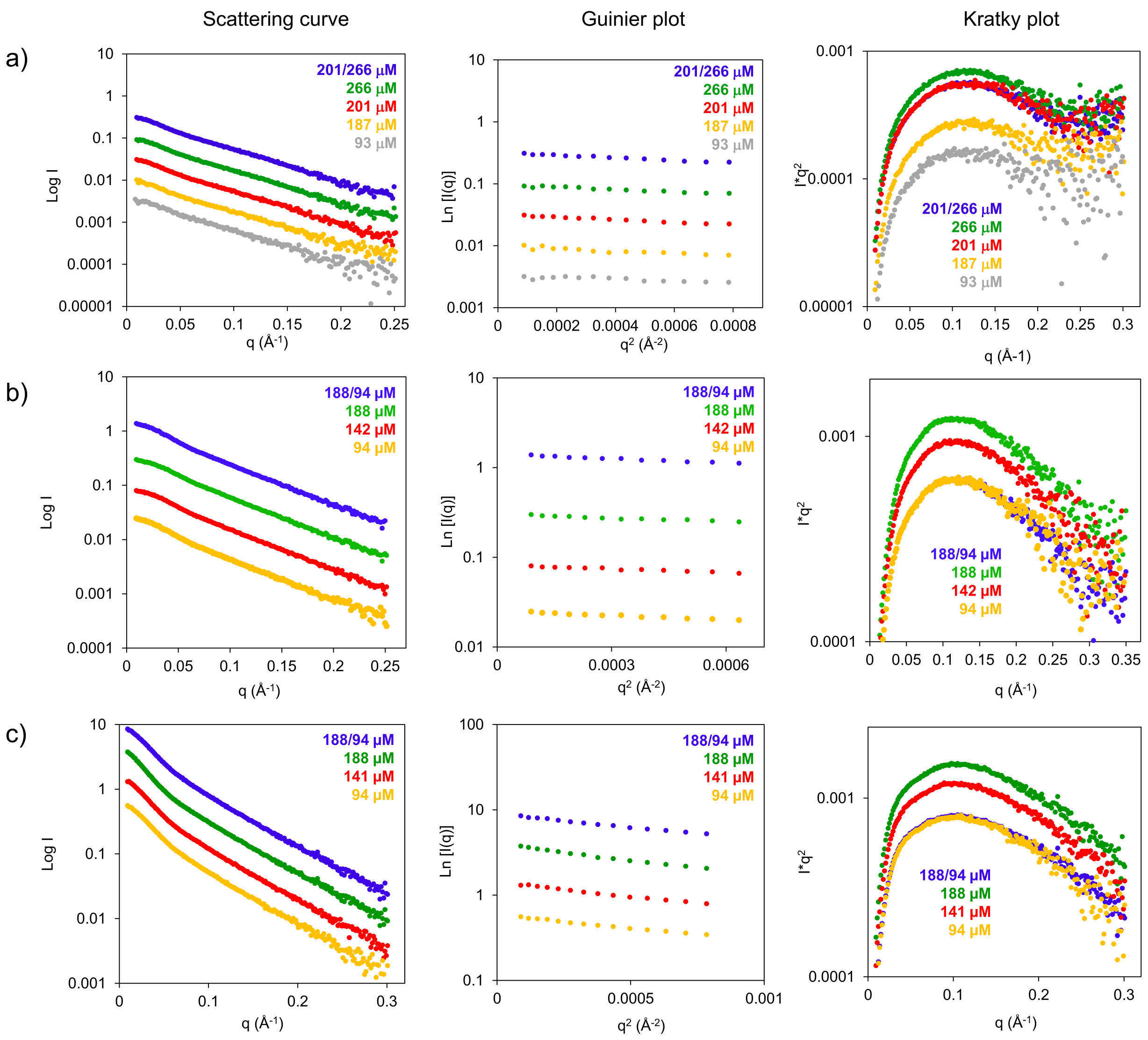

Figure S6. Scattering curves, Guinier and Kratky plots of A) XRCC1-CDB, **B)** 39 bp DNA and **C)** CDB-DNA complex.

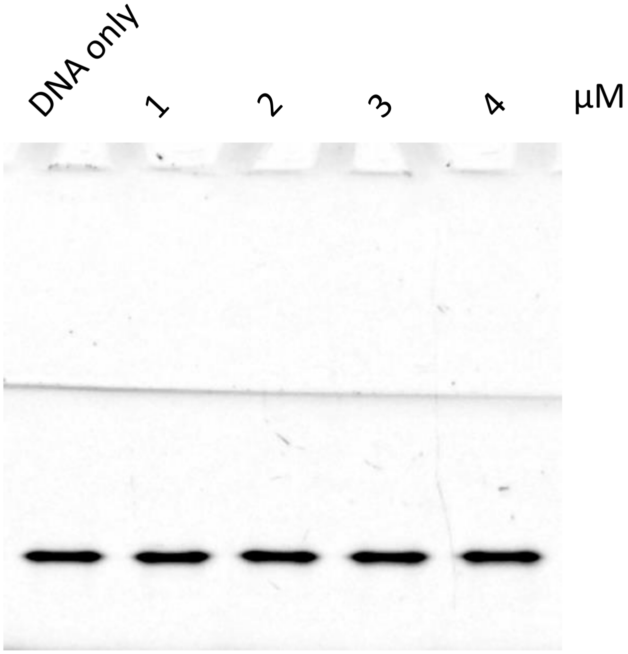

Figure S7. EMSA analysis of XRCC1$^{521\text{-}633}$ (BRCT2) DNA binding activity with 39 bp undamaged DNA. No binding of BRCT was observed at concentrations between 1-4 μM.

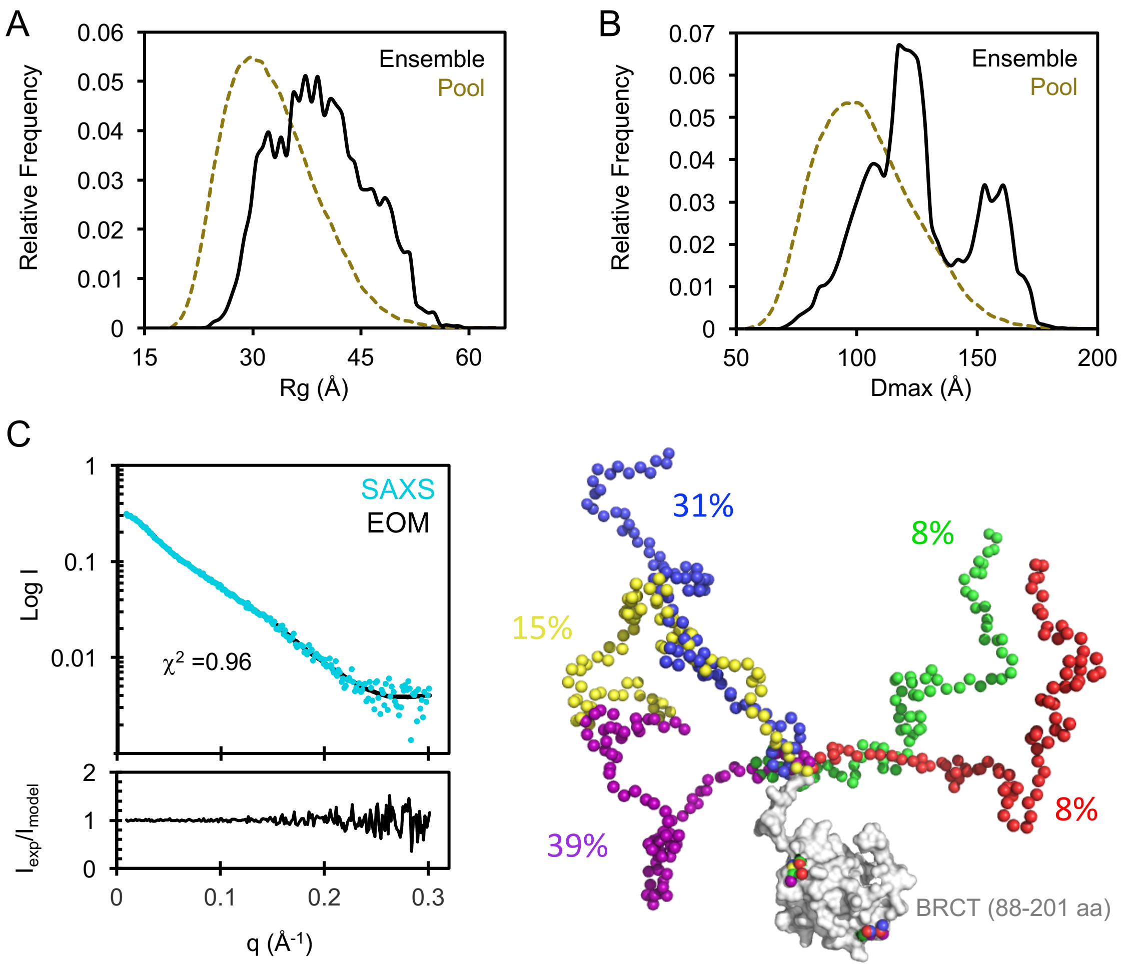

Figure S8. EOM analysis of XRCC1 CDB solution scattering. Comparison of radius of gyration, Rg **(A)** and maximum dimension, Dmax **(B)** distribution between the randomized pool (brown) and optimized ensemble (black). **(C)** Fitting of the theoretical solution scattering from the EOM ensemble (black) to experimental scattering of XRCC1 CDB (cyan). The best-fit ensemble generated with the available XRCC1 BRCT1 atomic structure (PDB ID 2D8M; grey) contains 5 conformers shown in red, green, blue, yellow and purple along with their percentage occurrence.

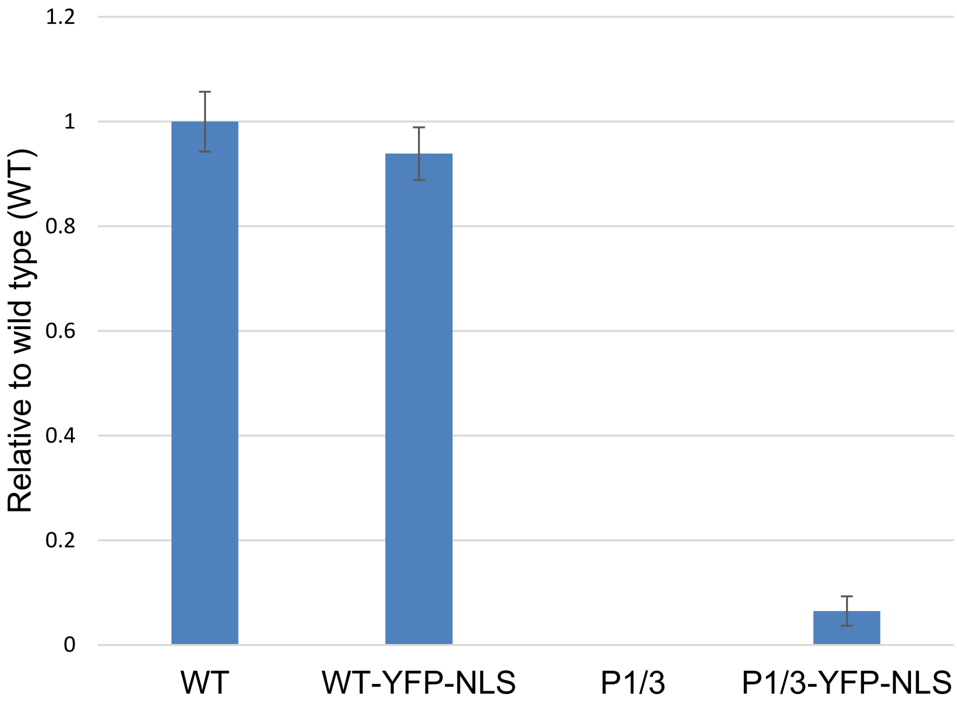

Figure S9. Comparison of DNA binding activity of WT XRCC1$^{219\text{-}633}$ and P1/3 mutant with added C-terminal YFP-NLS sequences. DNA binding was determined at 2 μM protein concentration. P1/3-YFP-NLS possessed ~ 6% of wild type DNA binding activity.

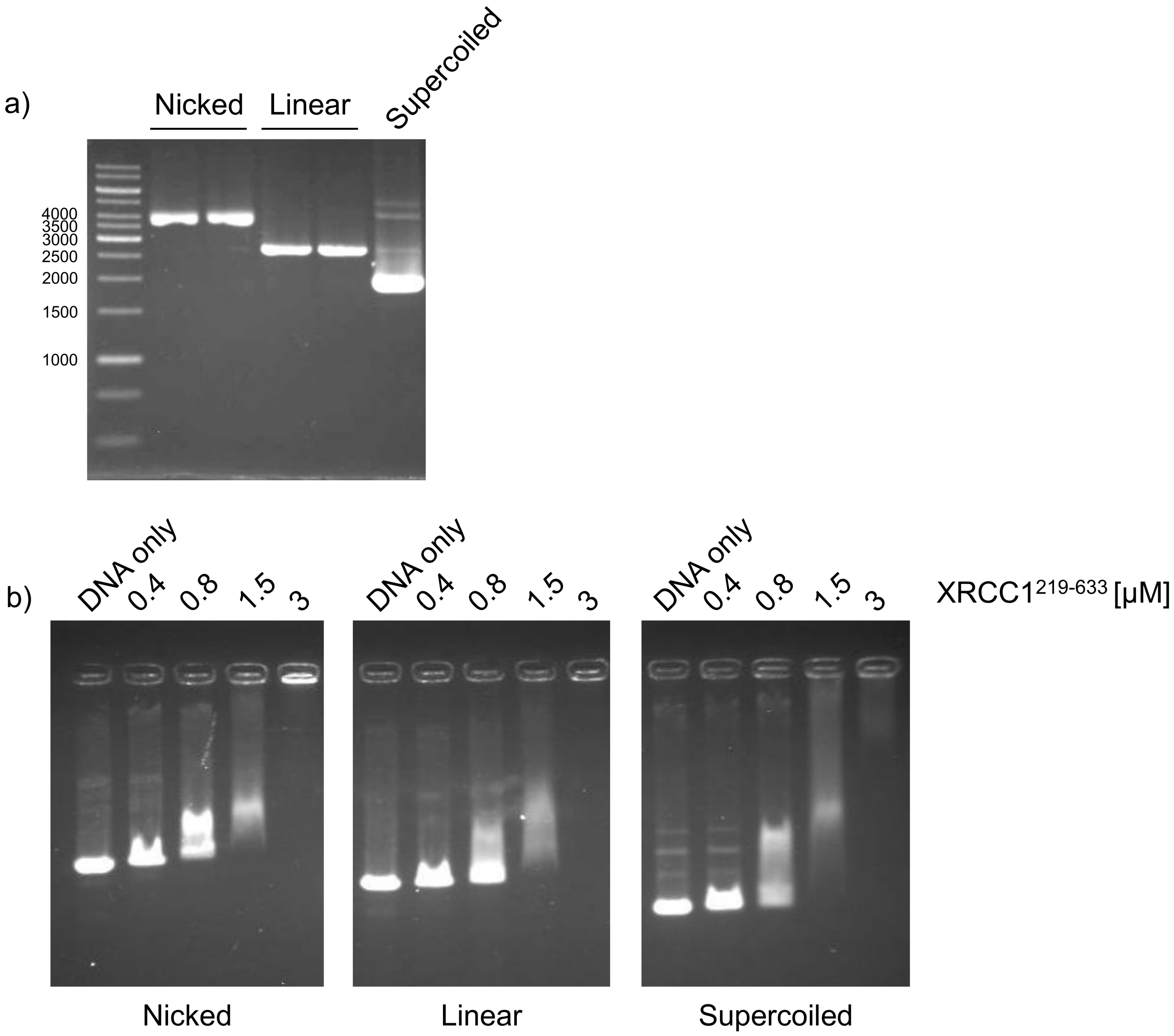

Figure S10. XRCC1$^{219\text{-}633}$ interacts with nicked, linear and supercoiled pUC19 plasmid. A) pUC19 plasmid (NEB) was digested with NtBspQI (NEB) or Nde1 (Thermo Scientific) to generate nicked and linear pUC19 respectively. Each digested product was purified using the PCR purification kit (Qiagen) to remove contaminants. **B)** EMSA of pUC19 substrates with XRCC1$^{219\text{-}633}$. DNA concentration was fixed at 20 nM for each reaction while XRCC1$^{219\text{-}633}$ concentration was varied. Protein and DNA were incubated in 20 mM Tris pH 8, 100 mM KCl, 1 mM DTT, 10 ug/uL BSA and 5% glycerol at room temperature for 1 hour before running on 1% TBE agrose gels. Gels were stained with ethidium bromide.

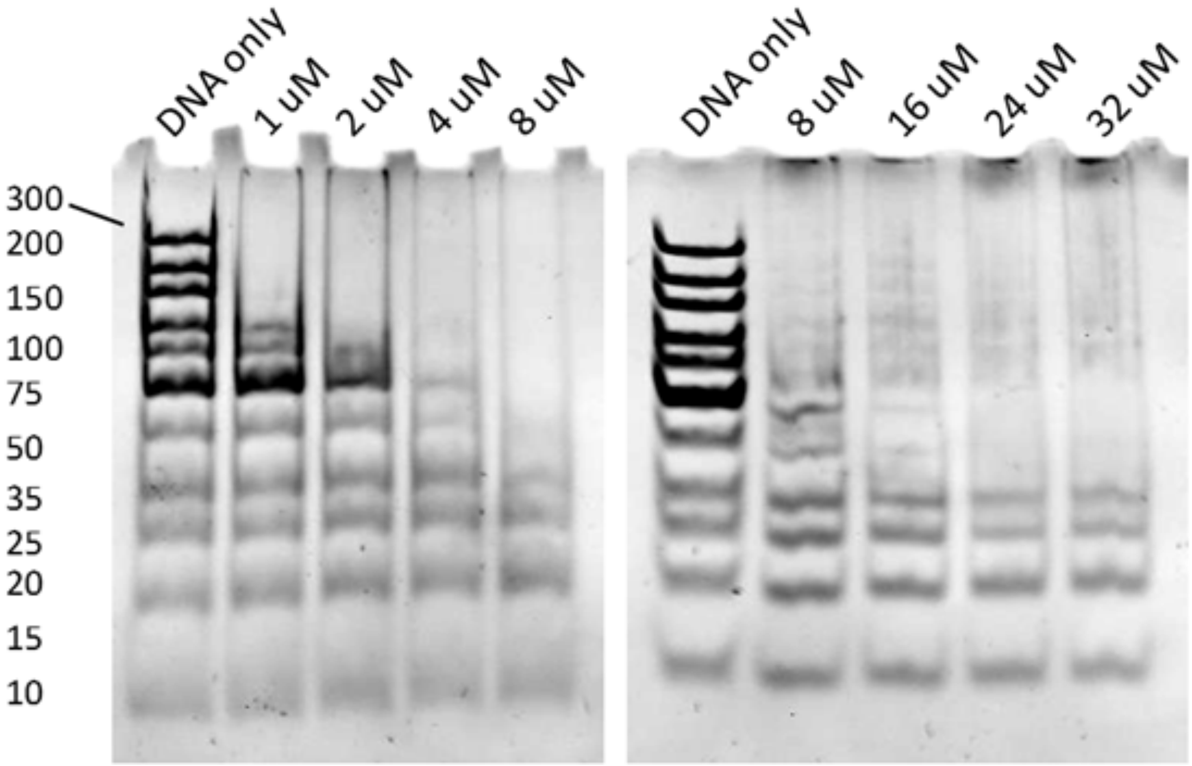

Figure S11. Analysis of XRCC1 DNA size binding preference. An electrophoretic mobility shift analysis was performed using a DNA low molecular weight ladder (FroggaBio) and increasing concentrations of XRCC1. Even at the highest concentration tested, XRCC1 failed to bind DNA smaller than 35 bp.

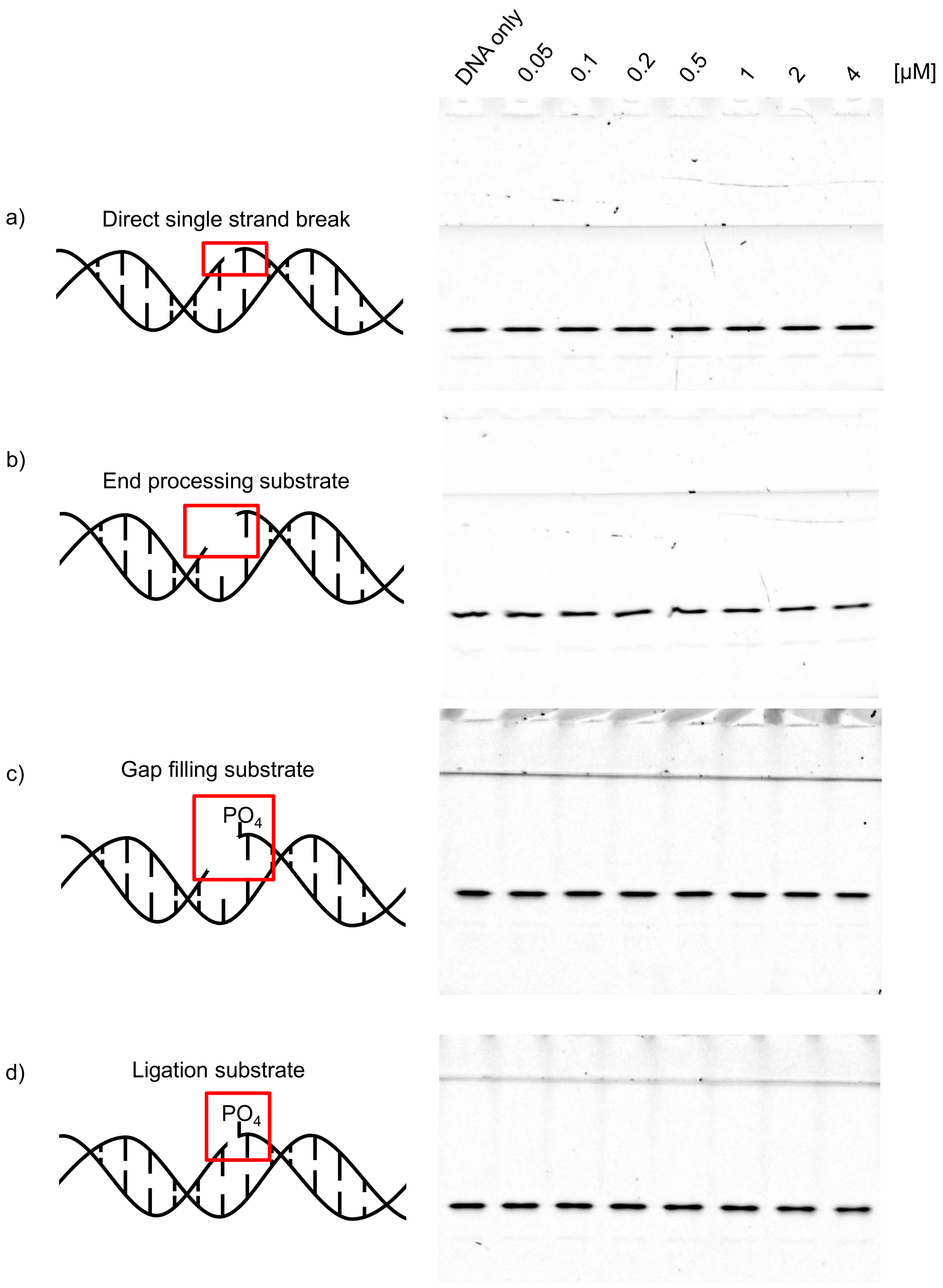

Figure S12. EMSA analysis of NTD (XRCC1[1-183]) interaction with different 39 bp DNA substrates. A-D) Different substrates reflecting multiple type of DNA potentially encountered by XRCC1 during repair. No interaction was detected for any DNA substrates tested.

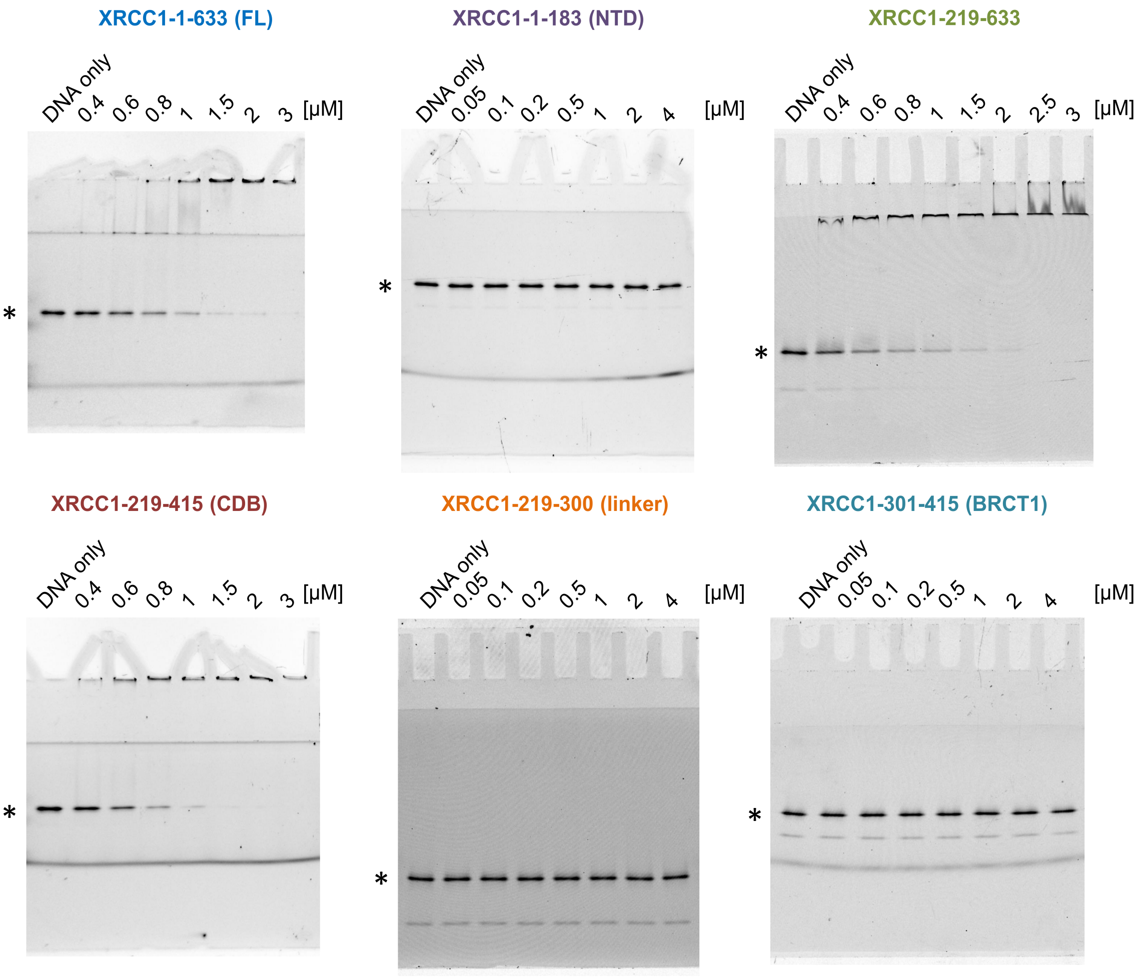

Figure S13. Full length gels from Figure 1. Asterisk indicates 39 bp DNA substrate.

Table S1. Summary of SAXS data analysis.

	CDB	DNA (39 bp)	CDB-DNA
**Data collection**			
Exposure time (min)	120	120	120
Concentration (μM)	266	94/188	94/188
**Structural parameters**			
Io	0.38	0.35	0.86
Io/conc	0.07	0.15	0.22
Rg (Å)	37.9	35	45.6
Dmax (Å)	130	120	155
$MW^{Experimental}$ (kDa)	25.8	23.9	59.9
$MW^{Calculated}$ (kDa)	21.4	24	45.4
